# Drugs and Clinical Approaches Targeting the Antiapoptotic Protein: A Review

**DOI:** 10.1155/2019/1212369

**Published:** 2019-09-29

**Authors:** Zeping Han, Jiening Liang, Yuguang Li, Jinhua He

**Affiliations:** ^1^Department of Laboratory Medicine, Central Hospital of Panyu District, Guangzhou, Guangdong 511400, China; ^2^School of Chemistry and Molecular Biosciences, The University of Queensland, St. Lucia, QLD, Australia

## Abstract

B-cell lymphoma 2 (Bcl-2) is a regulator protein involved in apoptosis. In the past few decades, this protein has been demonstrated to have high efficacy in cancer therapy, and several approaches targeting Bcl-2 have been tested clinically (e.g., oblimersen, ABT-737, ABT-263, obatoclax mesylate, and AT-101). This review reports potential Bcl-2 inhibitors according to current information on their underlying mechanism and the results of clinical trials. In addition, the function and mechanisms of other potentially valuable Bcl-2 inhibitors that did not show efficacy in clinical studies are also discussed. This summary of the development of Bcl-2 inhibitors provides worthwhile viewpoints on the use of biomedical approaches in future cancer therapy.

## 1. Introduction

In current gene and cell immunotherapy techniques, cellular pathways are popular and effective targets for curing cancer. It is important to discover a switch or inhibitor of a cellular pathway that is expressed not only in leukemia but also in solid tumors because solid tumors remain a critical barrier to cell therapy due to their size and the protective effects of the microenvironment [1]. Therefore, the identification of cellular pathway inhibitors is a valuable research topic for destroying the tumor microenvironment from the inside.

In the last few decades, researchers have tried to induce tumor regression by blocking cellular pathways to enhance the efficiency of therapeutic treatments. One such candidate is B-cell lymphoma 2 (Bcl-2) protein, a potential inhibitor of apoptosis that belongs to the Bcl-2 family, members of which are involved in several cellular pathways such as DNA damage/p53 pathway, survival/NF-*κ*B pathway, estrogen pathway, STAT pathway, and PI-3 kinase/AKT pathway [2]. Bcl-2 was the first identified mammalian regulator of apoptosis [3] and consists of four conserved domains (BH4, BH3, BH1, and BH2), which differentiate it from other Bcl-2 family members, (e.g., Bim, Bid, Puma, Noxa, Bad, Hrk, Bmf, and Bik) [4]. Among these homology motifs, BH3, BH1, and BH2 are the most commonly targeted in clinical approaches.

## 2. Clinical Approaches

In the past 30 years, efforts have been made to identify methods to cure Bcl-2 protein-related diseases, including molecular antibodies, small molecule drugs, and antisense oligonucleotides. In currently reported cases, the efficiency and safety of some clinical drugs targeting Bcl-2 protein have been demonstrated in hematologic carcinoma.

**Figure 1:**
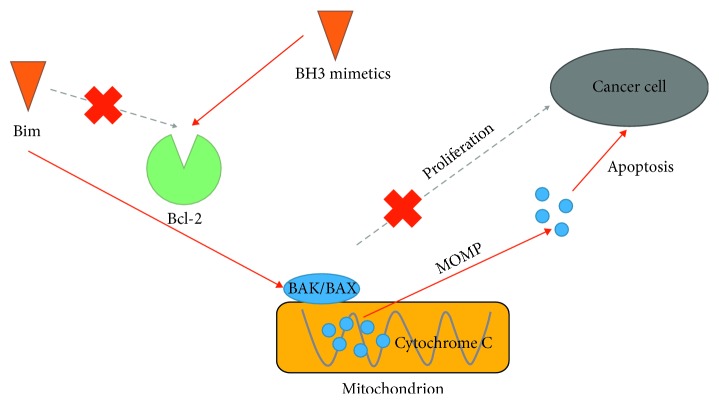
Bcl-2 inhibitors inducing apoptosis of cancer cells.

The mechanism of action of Bcl-2 inhibitors has been widely studied. Most Bcl-2 inhibitors block the binding of BH3-only proteins (mostly Bim) to Bcl-2 via a small molecule that mimics the BH3 domain. This process allows free Bim to activate Bak/Bax on the surface of mitochondria and induces mitochondrial outer membrane permeabilization (MOMP) to release cytochrome C, which leads to the death of cancer cells. The proliferation of specific cancer cells is also inhibited in the presence of cytochrome C [5] (Figure 1).

Oblimersen (G3139, Genasense) was the first developed drug that utilized Bcl-2 inhibitors and was produced by Genta Inc. It utilizes an 18-mer short sequence RNA to inactivate mRNA via hybridization to inhibit the production of Bcl-2 protein and the proliferation of lymphoma cells. Unfortunately, no difference was found in the 5-year survival rate of patients in phase III clinical trials of oblimersen between the test and reference groups, so oblimersen has been rejected twice for approval by the US Food and Drug Administration (FDA) [6]. Most clinical studies using only oblimersen ended before 2010, and it is unknown whether it decreased cancer cells by an antisense drug mechanism or the induction of interferon release via its own CpG motif. After the failure of single-use oblimersen, the antitumor efficacy of oblimersen combined with other drugs has been examined. Clinical trials of Bcl-2 inhibitors conducted from 2010 to 2019 are shown in Table 1. In 2010, Raab et al. reported a phase I trial of oblimersen combined with cisplatin and 5-fluorouracil in 15 patients with advanced esophageal, gastroesophageal junction, or gastric carcinoma, which resulted in 1 case with complete remission and 2 with a partial response [7]. In 2011, Galatin et al. indicated that 5 of 16 patients with refractory and advanced malignancies had stable disease after combined treatment with oblimersen and gemcitabine [8]. In 2013, Ott et al. reported that treatment of patients with advanced melanoma with oblimersen combined with temozolomide and albumin-bound paclitaxel resulted in 2 cases with complete remission, 11 with a partial response, and 11 with stable disease [9].

After oblimersen, Petros et al., comprising the Abbott team (Abbott Laboratories, USA), devised a new method called fragment-based drug discovery that could be used in the development of Bcl-2 inhibitors [29]. Initially, Abbott focused on Bcl-xL protein, but the high structural homology between Bcl-xL and Bcl-2 proteins (with a difference of only four amino acids in the active site) meant that the new drug was suitable for Bcl-2 and Bcl-xL [30]. Abbott divided the Bcl-xL binding site into two fragments, based on the previous discovery of two small chemical compounds (I and II) from a library of molecules specifically binding to the Bcl-xL BH_3_ domain. The study data indicated that modified compound I was able to enter the P2 binding pocket, while compound II could readily enter the P4 pocket [31, 32]. Combination of compounds I and II together with modified compound II resulted in an inhibitor with potent affinity [33]. Further developing the findings of Petros et al., an antibody targeting Bcl-2 protein appeared in 2005, when Oltersdorf et al. reported their candidate antibody ABT-737 and provided preclinical evidence for targeting Bcl-2 in treating solid tumors [34]. Even though this discovery was established using a mouse model, it led to the development of the orally administered drug navitoclax (ABT-263) by Tse et al. 3 years after the first report of ABT-737. Navitoclax showed effective tumor regression in patients with small-cell lung cancer and acute lymphocytic leukemia, but there was a large decrease in the number of platelets that encouraged the development of a new application with higher safety [35]. However, Zhang et al. suggested that Bcl-xL is crucial for the survival of mature platelets *in vivo*, so the previous two applications are not suitable drugs for patients [36]. Reports from 2012 and 2015 also confirmed the low efficacy of navitoclax [10–16]. AbbVie pharmaceuticals then introduced venetoclax (ABT-199) targeting BH3-binding sites only on Bcl-2 that maintains efficiency while protecting the survival of platelets, and improved treatment of chronic lymphocytic leukemia was reported [37]. In 2016, the FDA approved the use of venetoclax. In recent years, venetoclax has been tried in different combinations with other antitumor monoclonal antibodies or small molecule drugs; however, these attempts have mostly focused on hematologic carcinoma [17–23].

Obatoclax mesylate (GX15-070), another drug targeting the Bcl-2 family, has also shown efficacy in phase III clinical trials. This drug is a BH3 mimetic, so it can potentially bind to most Bcl-2 family members (including Bcl-2 protein). However, the main mechanism of this inhibitor is binding to Mcl-1 as a supplemental therapy in Bcl-xL^low^ or Bcl-xL^−^ cancer cells or cells with resistance to Bcl-2 inhibitors [38]. Bcl-2 protein is reportedly crucial for sustaining hematopoietic stem cells, but this antiapoptotic factor is also expressed at a high level in acute myeloid leukemia and acute lymphocytic leukemia [39]. Therefore, obatoclax mesylate provides an additional option for patients who need a Bcl-2 inhibitor. Unfortunately, the developers of obatoclax mesylate suddenly terminated their clinical trials in 2012, and no further details have been released since 2014 [24, 25].

AT-101 is an orally active pan-Bcl-2 inhibitor that consists of gossypol, a natural compound derived from the cotton plant [40]. In preclinical trials, it induced a strong apoptotic response in leukemic cells [41]. However, phase II trial data of AT-101 posted in 2010 indicated that it did not generate the expected response (NCT00286780), so the company stopped the clinical development of this drug until the founders decided to continue research in China. Recently, clinical trials using AT-101 in combination with other drugs for the treatment of solid tumors have been reported, such as its combination with docetaxel to treat head and neck cancer [27], in combination with docetaxel and prednisone to treat castration-resistant prostate cancer [42], in combination with topotecan to treat small-cell lung cancer [26], in combination with paclitaxel and carboplatin for different carcinoma types [43], and in combination with cisplatin and etoposide for extensive-stage small-cell lung cancer [28]. Given the reported findings, it is reasonable to believe that AT-101 could be a potent inhibitor of Bcl-2.

## 3. Other Valuable Candidates

In 2007, Mohammad et al. introduced another Bcl-2 inhibitor named TW-37, which is a small molecule inhibitor of Bcl-2/Bcl-XL/MUC-1 that could prevent Bcl-2 overexpression [44]. TW-37 has a higher affinity and selectivity for Bcl-2 compared with Bcl-xL protein. According to *in vitro* tests, TW-37 acts in lymphoma cells from patients, but not in normal peripheral blood cells [44]. In a mouse model, the combination of TW-37 with an MEK inhibitor was found to prevent the growth of melanoma cells [45]. Considering the features of TW-37, it should be a potent drug for inhibiting Bcl-2; however, due to unknown reasons, this inhibitor was not examined in clinical trials. Besides TW-37, several Bcl-2 inhibitors are in the preclinical development stage, such as HA14-1, sabutoclax, S55746 (S055746, BCL201), and gambogic acid. Among these compounds, S55746 shows similar high affinity to Bcl-2 as ABT-737 and ABT-199 [46].

## 4. Conclusion

With regard to Bcl-2 protein inhibitors, two main streams have been utilized in clinical approaches, namely, antibodies and small molecule drugs. Techniques have been developed to inhibit the expression and function of Bcl-2 and its family proteins at the gene and protein levels. According to current data, approximately 19 different Bcl-2 inhibitors are the subjects of preclinical or clinical studies and various combinations of therapeutic methods are being assessed for Bcl-2-positive cancer. Results from the clinical trials conducted in the last few decades suggest that the combination of Bcl-2 inhibitors with other antitumor drugs or RNA/DNA inhibitors will be more effective than their single use.

## Figures and Tables

**Table 1:** 2010–2019 Clinical trials of Bcl-2 inhibitors.

Bcl-2 inhibitor	Combined therapy with other drugs	Targeted cancer type	No. of patients	Complete remission	Partial response	Stable disease	Phase	Researchers
Oblimersen	Cisplatin and 5-fluorouracil	Advanced esophageal, gastroesophageal junction, and gastric carcinoma	15	1 (metastatic gastric carcinoma with pulmonary metastases)	2 (1 gastric carcinoma, 1 squamous cell carcinoma of the esophagus)		I	Raab et al. [7]
	Gemcitabine	Refractory and advanced malignancies	16	0	0	5	I	Galatin et al. [8]
	Temozolomide and albumin-bound paclitaxel	Advanced melanoma	32	2	11	11	I	Ott et al. [9]

Navitoclax (ABT-263)	None	SCLC or pulmonary carcinoid	47	0	1 (SCLC)	8 (5 SCLC, 3 atypical pulmonary carcinoid)	I	Gandhi et al. [10]
	None	Relapsed SCLC	39	0	1	9	II	Rudin et al. [11]
	None	Relapsed or refractory CLL	29	0	9	7	I	Roberts et al. [12]
	Irinotecan	Advanced solid tumors	31	0	2 (1 Merkel cell carcinoma, 1 colon carcinoma)	6	I	Tolcher et al. [13]
	Erlotinib	Advanced solid tumors	11	0	0	3	I	Tolcher et al. [14]
	Rituximab	Relapsed or refractory CD20^+^ lymphoid malignancies	29	6 (1 diffuse large B-cell lymphoma, 5 follicular lymphoma)	10 (5 CLL/SLL, 4 follicular lymphoma, 1 lymphoma/Waldenström's macroglobulinemia)		I	Roberts et al. [15]
	Rituximab	Previously untreated B-cell CLL	78	2	47	25	II	Kipps et al. [16]

Venetoclax (ABT-199)	Obinutuzumab	CLL and coexisting conditions	216	107	76		III	Fischer et al. [17]
	Ibrutinib	CLL	80	25	1		II	Jain et al. [18]
	Ibrutinib	CLL	91	8	48	22	II	Jones et al. [19]
	None	Relapsed or refractory non-Hodgkin lymphoma	106	14	33	32	I	Davids et al. [20]
	None	Acute myelogenous leukemia	32	6 (2 CR, 4 CRi)	0	6	II	Konopleva et al. [21]
	Decitabine or azacitidine	Acute myeloid leukemia	57	35	1	0	I	DiNardo et al. [22]
	Bendamustine and obinutuzumab	CLL	66	5	55		II	Cramer et al. [23]

Obatoclax mesylate (GX15-070)	Bortezomib	Relapsed or refractory mantle cell lymphoma	13	3	1	6	I/II	Goy et al. [24]
	None	Myelodysplastic syndromes with anemia or thrombocytopenia	24	0	0	17	II	Arellano et al. [25]

AT-101	Paclitaxel and carboplatin	Several types of solid tumors (most common tumor type was prostate, *N* = 11)	24	1 (esophageal cancer)	4 (1 NSCLC, 3 prostate)		I	Stein et al. [26]
	Docetaxel	Recurrent, locally advanced, or metastatic head and neck cancer	22	0	3	12	II	Swiecicki et al. [27]
	Cisplatin and etoposide	Extensive-stage SCLC	27	0	10	11	I	Schelman et al. [28]

CLL, chronic lymphocytic leukemia; CRi, complete response with incomplete blood count recovery; NSCLC, non-small-cell lung cancer; SCLC, small-cell lung cancer; and SLL, small lymphocytic leukemia.
